# Positive associations between different circulating trans fatty acids (TFAs) and urinary albumin excretion among adults in the U.S.: a population-based study

**DOI:** 10.1186/s12944-023-01917-w

**Published:** 2023-09-14

**Authors:** Yuancheng Zhou, Chengcheng Wei, Xincheng Gao, Yi Sun, Xiaomin Han

**Affiliations:** Department of Urology, Union Hospital, Tongji Medical College, Huazhong University of Science and Technology, 1277 Jiefang Road, Wuhan, 430022 Hubei Province China

**Keywords:** Trans-fatty acids, Albumin-creatinine ratio, NHANES, Cross-sectional study, American adult

## Abstract

**Background:**

It is well established that the consumption of trans-fatty acids (TFAs) can increase the incidence of total mortality, cardiovascular disease, cancer, and diabetes. However, there are still no demographic studies on the effects of circulating TFA isoforms on the albumin-creatinine ratio (ACR), an early marker of chronic kidney disease. Our goal was to explore the possible relationships between TFAs and ACR.

**Methods:**

In this study, complete TFAs and urinary ACR data were collected from the National Health and Nutrition Examination Survey (NHANES) (2009–2010 and 1999–2000 cycles). The independent linear relationships between different circulating TFA isoforms and the ACR were examined by performing multivariable linear regression models. Machine learning was used to analyze the contribution of the different TFA isoforms to the ACR. To assess the nonlinearity of the relationship, smooth curve fitting and an analysis of threshold effect were performed, and a stratified analysis was conducted to identify possible susceptible populations.

**Results:**

Our analysis included a total of 3785 individuals. Elaidic acid, linolelaidic acid, and sum TFAs were shown to be positively associated with the ACR after full adjustment by weighted multivariable regression analysis. In the subgroup analysis, the positive associations were maintained in participants with hypertension and without diabetes. In the XGBoost model of the ACR, Sum TFAs were found to be the most crucial factor. In addition, smooth curve fitting showed that there was a nonlinear relationship between the different TFAs and the ACR, and there was a saturation point.

**Conclusions:**

Our study demonstrated that TFA isoforms were positively and independently correlated with urinary albumin excretion, especially in participants with hypertension and without diabetes. This suggested that reducing trans fatty acid intake may reduce the risk of renal events.

**Supplementary Information:**

The online version contains supplementary material available at 10.1186/s12944-023-01917-w.

## Introduction

Albuminuria is becoming a significant public health concern because it has a high prevalence, ranging from 5 to 19%, in the general population [1]. Microalbuminuria is reported to occur at even higher rates in patients with hypertension and diabetes. Increased urinary albumin excretion serves as an indicator not only for early-stage kidney disease but also as an independent predictor for the related risk of cardiovascular events (CV), as well as chronic kidney disease (CKD) [2–4]. Given the negative impact of albuminuria on clinical outcomes, it warrants close attention from healthcare professionals. An efficient and convenient method for assessing and defining albuminuria is the randomized urinary albumin-creatinine ratio (ACR), which could eliminate confounders such as food and exercise [5, 6]. Additionally, the calculation of ratios improves sensitivity and corrects for changes in hydration status, independent of dilution or urine concentration [7].

Trans fatty acids (TFAs), a specific type of unsaturated fatty acid, include ruminant TFAs and industrial TFAs. Enzymes create them in the rumen of animals during hydrogenation, and they are also produced through the partial hydrogenation of vegetable oils (PHVO) [8]. The primary sources of TFA exposure for people are industrially processed high-fat foods (e.g., chicken cutlets, fries, burgers, margarine, and its products) and animal products (e.g., meat) [9]. Up to 50% of the TFAs are present due to PHVO in industrially-processed foods, with elaidic acid being the predominant isoform. In contrast, the TFA content of ruminant fats is generally low (1–8%) [10]. It is well established that the consumption of TFAs can increase the incidence of total mortality, cardiovascular disease, cancer, and diabetes [11–13]. There is also evidence that different TFA isoforms have different metabolic and physiological effects on various pathologies [14]. A previous study demonstrated that elevated levels of circulating elaidic acid were linked to a higher occurrence of dementia [15], and linoelaidic acid was shown to be the TFA isoforms that was positively associated with total mortality, mainly due to the increased occurrence of coronary artery disease [12]. Therefore, according to the dietary guidelines from the World Health Organization, TFA intakes should be limited to less than 1% of total caloric intake to reduce the risk of chronic diseases [16].

Starting from 1999, the U.S. FDA has required that the amount of TFAs per serving should be displayed on packaged foods, and the TFA consumption declined from 2 to 3% in the early 1990s to approximately 1% in 2009–2010 [17, 18]. However, TFAs are still used at high levels in low- and middle-income countries. In most surveys, trans-fat intakes were estimated by using dietary questionnaires and may be limited by residual confounding of unmeasured ingredients, misclassification, or imprecise assessment, particularly the inability to identify the effects of specific TFA isoforms [13]. Blood biomarkers are relatively sensitive and stable indicators to assess exogenous intake. Circulating fatty acids could reflect dietary intake over the past 6–12 weeks [19]. As an alternative, blood TFA levels may serve as a preferred biomarker of dietary TFA intake, establishing a stronger relationship with adverse outcomes [20]. To our knowledge, there are no demographic studies on the effects of circulating TFA isoforms on ACR. In the present study, a secondary analysis was conducted based on the U.S. NHANES to determine whether TFA isoforms are associated with the ACR. The results will help to enhance the understanding of the biological effects of TFA isoforms on urinary albumin excretion and thus provide information for the appropriate intake of TFAs in the daily diet.

## Materials and methods

### Data availability

The Prevention National Health Statistics Center and National Centers for Disease Control (CDC) have undertaken an important initiative known as the NHANES since 1960, which was conducted sporadically between 1960 and 1994. Since 1999, it has been conducted continuously. Data are released in 2-year cycles. The NHANES program collects information on possible health risk factors and nutritional status among noninstitutionalized civilians in the United States. The information and the methodology underlying this study are highly detailed and can be found on the NHANES website (https://wwwn.cdc.gov/nchs/nhanes/analyticguidelines.aspx) [21, 22]. The demographic variables dataset, blood pressure dataset, albumin & creatinine–urine dataset, cholesterol–HDL dataset, cholesterol-LDL & triglycerides dataset, cholesterol-total dataset, glycohemoglobin dataset, standard biochemistry profile dataset, trans fatty acids, alcohol use dataset, physical activity dataset, smoking-cigarette use dataset and medical conditions dataset were selected. The National Center for Health Statistics research ethics review board conducted a comprehensive assessment and evaluation of the NHANES study prior to approving, and the study was designed and conducted in accordance with the Helsinki Declaration of the World Medical Association [21] (https://www.cdc.gov/nchs/nhanes/irba98.htm#print).

### Study population

To assess the participants’ medical and physiological conditions, they underwent standard home interviews, followed by health checkups at mobile screening centers, and collected laboratory data through various laboratory tests. All the data were collected from NHANES (2009–2010, 1999–2000) owing to their complete variables for calculating TFAs, ACR, and eGFR using the same protocol. It initially included 20,502 participants. Our analysis excluded samples based on the following criteria: (1) aged < 20 years old (n = 9404), (2) pregnant (n = 323), (3) missing complete data about ACR (n = 1406), TFAs (n = 5535) and eGFR (n = 49). Eventually, in our final analysis, we included 3785 eligible participants.

### TFAs measurement

According to a standardized protocol, blood samples are taken from participants’ veins during the morning fast. Gas chromatography-mass spectrometry (GC/MS) is employed to measure the free and esterified levels of sorted TFAs (palmitelaidic acid, linoelaidic acid, vaccenic acid, elaidic acid) in plasma samples. Lagerstedt et al. have described the measurement method [23]. All analytical quality control procedures follow a comprehensive data quality assurance program. In addition, based on the following formula, TFAs were summed: Sum TFAs = vaccenic acid + linoelaidic acid + palmitelaidic acid + elaidic acid.

### ACR measurement

In a standardized mobile examination center, blood as well as urine samples were collected from NHANES participants. Urinary albumin and creatinine levels are determined by analysis of individual spotted urine samples by a modified Jaffe kinetic method and solid-phase fluorescent immunoassay. The ACR (mg/g) was calculated by dividing the urinary albumin by the urinary creatinine. Albuminuria was regarded as the outcome variable in our analysis. Normal albuminuria was defined as ACR less than 10 mg/g, mildly increased albuminuria was defined as ACR between 10 and 30 mg/g, and moderately increased albuminuria was defined as ACR equal to or greater than 30 mg/g [24].

### Covariates

Based on a previous article, the study also included covariates that might influence the correlation between TFAs and ACR [25–29]. The following covariates are as follows: Sociodemographic variables included age, gender, education level, race, and poverty income ratio. Variables of laboratory data included ALT (IU/L), AST (IU/L), SCr (µmol/L), total cholesterol (mmol/L), triglycerides (mmol/L), HDL-C (mmol/L), LDL-C (mmol/L), serum albumin (g/L), serum uric acid (µmol/L), glycohemoglobin (%), eGFR (ml/min/1.73m^2^). Personal life history and physical examination data included BMI (kg/m^2^), SBP (mmHg), DBP (mmHg), waist circumference (cm), physical activity (MET-based rank) (%), had at least 12 alcohol drinks/1 year (yes/no), do you now smoke cigarettes (yes/no), now taking prescribed medicine for HBP (yes/no), now taking prescribed medicine for high cholesterol level (yes/no). Finally, the analyzed samples’ comorbidities data included hypertension history (yes/no), non-alcoholic fatty liver disease (NAFLD, yes/no), and diabetes history (yes/no/borderline). MET values, activity type, weekly frequency, and duration are all factors that can be used to determine physical activity (PA). The formula follows PA (MET-h/wk) = MET × weekly frequency × duration of each physical activity. Finally, PA was categorized as high-intensity group (> 50MET-h/wk), low-intensity group (1-50MET-h/wk), and no physical activity group (< 1MET-h/wk) [30]. eGFR of every participant was calculated based on the CKD Epidemiology Collaboration (CKD-EPI) creatinine equation [31]. Serum ALT identifies individuals suspected of NAFLD, and is often used as a monitoring biomarker and a screening test for NAFLD [32, 33]. Suspected NAFLD individuals were assumed to have serum ALT > 19 IU/L in women and > 30 IU/L in men, and they were required to have no significant alcohol intake or any other known factors causing liver disease [33].

### Statistical analysis

The CDC guidelines’ criteria were used to conduct a statistical analysis of the TFAs and the ACR. The NHANES sampling weights were constructed for combined survey cycles. We utilized Trans Fatty Acid Subsample 2 Year Weight (WTTFA2YR) for weighted analysis because TFA data were collected from those sample persons who were subsampled to those who were detected in the mobile examination center (MEC). Additionally, information about the first-stage sampling procedure (i.e., the strata and primary sampling units) was used to analyze complex survey data to estimate the variance properly. Continuous variables and categorical parameters are represented by the means with standard errors (SE) and percentages or frequencies, respectively. Because TFAs have a skewed distribution, they were log_2_-transformed when conducting the regression analysis. Initially, a chi-square test, which is employed for categorical variables, or an ANOVA test, which is utilized for continuous variables, was performed to examine the difference among the individuals by sum TFA groups and ACR stages. Second, the associations between the different TFAs and the ACR were detected using multivariable weighted linear regression in three different models. Model 1 was adjusted for none. Model 2 was adjusted for age, gender, education level, race/ethnicity, and poverty-to-income ratio. Model 3 was adjusted for age, gender, education level, race/ethnicity, poverty income ratio, ALT, AST, SCr, serum uric acid, total cholesterol, LDL-C, HDL-C, triglycerides, serum albumin, glycohemoglobin, eGFR, BMI, SBP, DBP, waist circumference, physical activity (MET-based rank), current cigarette use, now taking prescribed medicine for HBP, now taking prescribed medicine for high cholesterol level, had at least 12 alcohol drinks/1 year, hypertension history, NAFLD, coronary heart disease and diabetes history. Third, the continuous variables of the different TFAs were divided into four quartile ratios. The multiple linear regression models, including Models 1, 2, and 3, were constructed to determine the relationships between the different TFAs and the ACR. Fourth, stratified factors were used to analyze the associations between different TFAs and the ACR in subgroups, including age, BMI, eGFR, hypertension, diabetes, NAFLD, and coronary heart disease. Additionally, a term of interaction was incorporated to assess the heterogeneity of relationships among the various subgroups. Next, the XGBoost algorithm model for machine learning was used to analyze the contribution (gain) of the different TFAs to the ACR. Finally, a generalized additive model (GAM) was used to construct a smooth curve fitting, which was performed to determine the nonlinear associations between the different TFA isoforms and the ACR. Moreover, the saturation value between TFAs and the ACR was detected by a threshold effects analysis model. To avoid bias, we used multiple imputations for missing data [34]. Five replications and chain equations were created by using the MICE package [35]. Furthermore, the robustness of the results was assessed through the utilization of sensitivity analysis. First, to eliminate the effect of decreasing TFA intake over 10 years, the relationship between TFAs (divided into four quartile ratios) and the ACR was analyzed separately for the two NHANES survey cycles of 2009–2010 and 1999–2000. Second, to eliminate extrema’s potential effects, we excluded the data for the last 5% of TFA concentrations and analyzed them by using the smooth curve fitting. The results of the multivariate analysis are derived from the computed dataset and adhere to Rubin’s rules. All analyses were conducted using R version 4.0.2 (http://www.R-project.org, The R Foundation) and Empower software (www.empowerstats.com; X&Y Solutions, Inc., Boston, MA). P value of less than 0.05 was considered statistically significant.

## Results

### Characteristics of participants enrolled

**Fig. 1:**
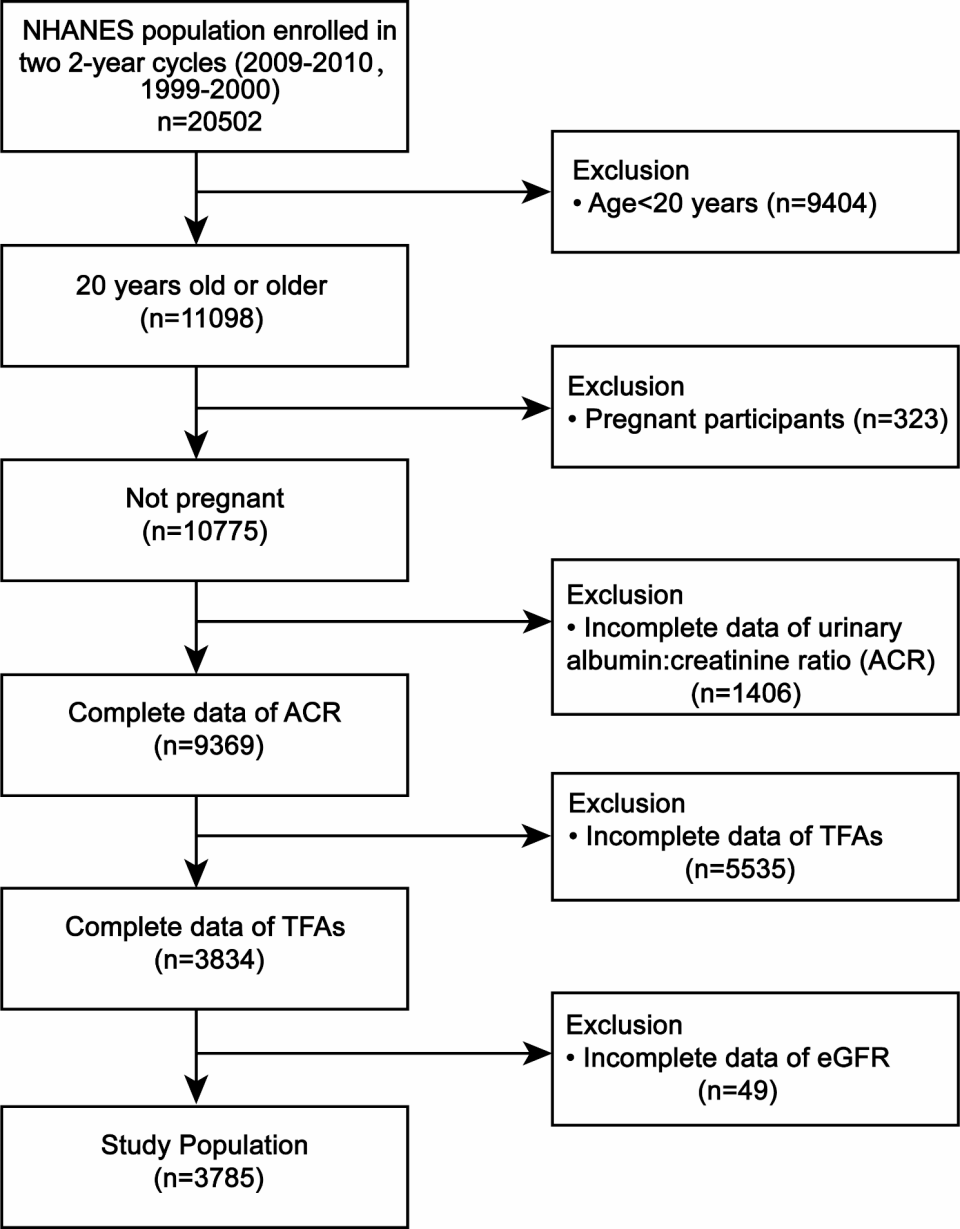
Flowchart of the study design and participants. NHANES, National Health and Nutrition Examination Survey; ACR, albumin-creatinine ratio; TFAs, trans-fatty acids; eGFR, estimated-glomerular filtration

Figure 1 illustrates the process of selecting the participants. A total of 3785 participants with complete data included in this study. The distribution of characteristics from NHANES (2009–2010, 1999–2000) for selective participants grouped by different sum TFAs (quartiles, Q1–Q4) is shown in Table 1. Palmitelaidic acid, vaccenic acid, elaidic acid, and linolelaidic acid showed distribution differences with statistical significance (all *P* < 0.05). Furthermore, subjects with increased sum TFAs had reduced PA, alcohol drinks, and proportion of prescribed medications, elevated age, total cholesterol, triglyceride, LDL-C, uric acid, serum albumin, urinary albumin, urinary creatinine, ACR, BMI, SBP, DBP, waist circumference, and decreased AST, SCr, HDL-C in our study. Compared with the various groups, there is no statistical significance observed in gender, poverty-to-income ratio, ALT, eGFR, hypertension history, NAFLD, coronary heart disease, and diabetes history. Most participants were non-Hispanic white individuals, and the following were Mexican American.

**Table 1:** characteristics of the study population

	Q1	Q2	Q3	Q4	P-value
N	945	946	947	947	
**Sociodemographic variables**					
Age (years)	46.44 ± 0.56	49.20 ± 0.57	51.58 ± 0.57	51.81 ± 0.58	< 0.001
Gender					0.828
Male	466 (49.31%)	457 (48.31%)	461 (48.68%)	447 (47.20%)	
Female	479 (50.69%)	489 (51.69%)	486 (51.32%)	500 (52.80%)	
Race/Ethnicity (%)					< 0.001
Mexican American	155 (16.40%)	196 (20.72%)	239 (25.24%)	255 (26.93%)	
Other Hispanic	112 (11.85%)	100 (10.57%)	82 (8.66%)	45 (4.75%)	
Non-Hispanic White	396 (41.90%)	441 (46.62%)	461 (48.68%)	516 (54.49%)	
Non-Hispanic Black	197 (20.85%)	177 (18.71%)	150 (15.84%)	111 (11.72%)	
Other race/ethnicity	85 (8.99%)	32 (3.38%)	15 (1.58%)	20 (2.11%)	
Education level (%)					< 0.001
Less than high school	244 (25.82%)	282 (29.81%)	347 (36.64%)	330 (34.85%)	
High school or GED	172 (18.20%)	216 (22.83%)	213 (22.49%)	244 (25.77%)	
Above high school	529 (55.98%)	448 (47.36%)	387 (40.87%)	373 (39.39%)	
Poverty to income ratio	2.57 ± 0.05	2.46 ± 0.05	2.46 ± 0.05	2.50 ± 0.05	0.443
**Variables of laboratory data**					
ALT (IU/L)	24.98 ± 0.57	27.45 ± 1.06	26.43 ± 0.77	27.39 ± 0.77	0.335
AST (IU/L)	25.87 ± 0.45	27.42 ± 0.97	25.98 ± 0.69	25.05 ± 0.55	< 0.001
SCr (µmol/L)	74.27 ± 0.61	73.78 ± 0.69	69.40 ± 0.70	65.96 ± 0.75	< 0.001
Total Cholesterol (mmol/L)	4.71 ± 0.03	5.10 ± 0.03	5.24 ± 0.03	5.52 ± 0.04	< 0.001
Triglyceride (mmol/L)	0.99 ± 0.01	1.33 ± 0.02	1.57 ± 0.03	2.25 ± 0.06	< 0.001
LDL-C (mmol/L)	2.77 ± 0.03	3.09 ± 0.03	3.22 ± 0.86	3.34 ± 0.03	< 0.001
HDL-C (mmol/L)	1.49 ± 0.01	1.39 ± 0.01	1.32 ± 0.03	1.19 ± 0.01	< 0.001
Serum uric acid (µmol/L)	315.67 ± 2.59	325.33 ± 2.76	326.96 ± 2.80	325.68 ± 2.84	0.018
Serum albumin (g/L)	42.77 ± 0.10	42.66 ± 0.10	43.44 ± 0.11	43.93 ± 0.10	< 0.001
Albumin, urine (mg/L)	11.94 ± 0.56	12.71 ± 0.64	13.28 ± 0.56	15.65 ± 0.71	< 0.001
Creatinine, urine (mg/dl)	125.69 ± 2.49	129.57 ± 2.54	133.88 ± 2.54	137.34 ± 2.72	0.009
Glycohemoglobin (%)	5.58 ± 0.02	5.62 ± 0.02	5.62 ± 0.02	5.70 ± 0.039	0.123
eGFR (ml/min/1.73 m^2^)	111.74 ± 1.07	109.44 ± 1.07	110.23 ± 1.06	110.28 ± 1.11	0.565
ACR (mg/g)	10.01 ± 0.38	10.22 ± 0.39	10.63 ± 0.39	12.06 ± 0.45	0.001
Palmitelaidic acid (µmol/L)	2.81 ± 0.03	4.19 ± 0.03	5.62 ± 0.04	9.19 ± 0.11	< 0.001
Vaccenic acid (µmol/L)	12.03 ± 0.10	19.70 ± 0.10	29.56 ± 0.16	55.59 ± 0.82	< 0.001
Elaidic acid (µmol/L)	9.04 ± 0.08	15.35 ± 0.10	24.40 ± 0.15	48.78 ± 0.69	< 0.001
Linolelaidic acid (µmol/L)	1.25 ± 0.01	1.69 ± 0.02	2.33 ± 0.02	3.73 ± 0.05	< 0.001
**Medical examination and personal life history**					
BMI (Kg/m^2^)	27.64 ± 0.21	29.26 ± 0.21	29.18 ± 0.21	29.08 ± 0.20	< 0.001
Waist circumference (cm)	95.06 ± 0.50	98.93 ± 0.51	99.00 ± 0.51	99.15 ± 0.49	< 0.001
SBP (mmHg)	120.9 ± 0.58	122.1 ± 0.55	125.3 ± 0.61	126.7 ± 0.62	< 0.001
DBP (mmHg)	67.9 ± 0.40	69.9 ± 0.39	70.3 ± 0.45	71.6 ± 0.45	< 0.001
Physical activity (MET-based rank) (%)					< 0.001
No physical activity	317 (33.54%)	353 (37.32%)	320 (33.79%)	288 (30.41%)	
Low-intensity physical activity	332 (35.13%)	335 (35.41%)	417 (44.03%)	462 (48.79%)	
High-intensity physical activity	296 (31.32%)	258 (27.27%)	210 (22.18%)	197 (20.80%)	
Had at least 12 alcohol drinks/1 year?					< 0.001
Yes	711 (75.24%)	698 (73.78%)	665 (70.22%)	621 (65.58%)	
No	234 (24.76%)	248 (26.22%)	282 (29.78%)	326 (34.42%)	
Current cigarette use					0.003
Every day	358 (37.88%)	367 (38.79%)	350 (36.96%)	359 (37.91%)	
Some days	97 (10.26%)	82 (8.67%)	53 (5.60%)	62 (6.55%)	
Not at all	490 (51.85%)	497 (52.54%)	544 (57.44%)	526 (55.54%)	
Taking prescribed medicine for HBP					< 0.001
yes	247 (26.14%)	260 (27.48%)	204 (21.54%)	198 (20.91%)	
No	698 (73.86%)	686 (72.52%)	743 (78.46%)	749 (79.09%)	
Taking prescribed medicine for high cholesterol level					0.002
Yes	203 (21.48%)	209 (22.09%)	161 (17.00%)	159 (16.79%)	
No	742 (78.52%)	737 (77.91%)	786 (83.00%)	788 (83.21%)	
**Comorbidities (%)**					
Hypertension history					0.136
Yes	278 (29.42%)	303 (32.03%)	326 (34.42%)	308 (32.52%)	
No	667 (70.58%)	643 (67.97%)	621 (65.58%)	639 (67.48%)	
NAFLD					0.348
Yes	101 (10.69%)	97 (10.25%)	99 (10.45%)	119 (12.57%)	
No	844 (89.31%)	849 (89.75%)	848 (89.55%)	828 (87.43%)	
Diabetes history					0.771
Yes	84 (8.89%)	70 (7.40%)	88 (9.29%)	80 (8.45%)	
No	848 (89.74%)	860 (90.91%)	842 (88.91%)	854 (90.18%)	
Border line	13 (1.38%)	16 (1.69%)	17 (1.80%)	13 (1.37%)	
Coronary heart disease					0.537
Yes	29 (3.07%)	28 (2.96%)	38 (4.01%)	35 (3.70%)	
No	916 (96.93%)	918 (97.04%)	909 (95.99%)	912 (96.30%)	

Q1–Q4: Grouped by quartile according to the sum of TFAs. Abbreviations: TFAs, trans fatty acids; GED, general educational development; ALT, alanine transaminase; AST, aspartate transaminase; SCr, serum creatinine; LDL-C, low-density lipoprotein; HDL-C, high-density lipoprotein; eGFR, estimated glomerular filtration rate; BMI, body mass index; SBP, systolic blood pressure;DBP, diastolic blood pressure; NAFLD, non-alcoholic fatty liver disease

### The regression analysis between TFAs and ACR

To clarify the relationship between TFAs and ACR, we constructed a weighted linear regression model (Table 2). Out of all the outcomes, we discovered a statistically significant positive correlation between elaidic acid, linolelaidic acid, sum TFAs, and ACR in all the models. In model 3, the positive association still remained stable, which indicates that the ACR increased by 0.74 mg/g (0.24, 1.24), 0.83 mg/g (0.15, 1.50) and 0.66 mg/g (0.11, 1.22) for each additional unit of log_2_-elaidic acid, log_2_- linolelaidic acid and log_2_-sum TFAs, respectively (all *P* < 0.05). Moreover, in our study, there was no significant relationship found between palmitelaidic acid, vaccenic acid and the ACR in model 3. Additionally, we transformed TFAs from continuous variables to categorical variables (quartiles) for sensitivity analysis and its calculated P for trend (Table 3). Compared with the lowest corresponding quartile, the ACR increased by 2.02 mg/g (0.76–3.28, *P* = 0.0017) in the highest elaidic acid quartile, and increased by 1.46 mg/g (0.19–2.73, *P* = 0.0244) in the highest sum TFAs quartile (all *P* < 0.05). Differences with no statistical significance were observed in the highest palmitelaidic acid quartile, vaccenic acid quartile and linolelaidic acid quartile compared to the lowest corresponding quartile. However, all P for trend values were less than 0.05. Moreover, we discovered the statistically significant inverse association between TFAs and eGFR in all models (Table S1), which could further confirm the adverse effect of TFAs on renal function.

**Table 2:** Multivariate weighted linear model analysis reveals the association between the log_2_-transformed TFAs and ACR

Exposure	Model 1 β, (95%CI), p	Model 2 β, (95%CI), p	Model 3 β, (95%CI), p
Palmitelaidic acid	1.15 (0.61, 1.70) < 0.001	0.62 (0.08, 1.17) 0.026	0.65 (-0.02, 1.31) 0.0555
Vaccenic acid	0.65 (0.20, 1.10) 0.005	0.40 (-0.05, 0.85) 0.083	0.43 (-0.09, 0.95) 0.1079
Elaidic acid	1.18 (0.76, 1.59) < 0.001	0.66 (0.24, 1.08) 0.002	0.74 (0.24, 1.24) 0.0039
Linolelaidic acid	1.29 (0.76, 1.83) < 0.001	0.80 (0.26, 1.34) 0.004	0.83 (0.15, 1.50) 0.0167
Sum TFAs	1.01 (0.55, 1.48) < 0.001	0.59 (0.12, 1.05) 0.014	0.66 (0.11, 1.22) 0.0198

Model 1: Non-adjusted model adjusts for none. Model 2: Minimally adjusted model adjusts for age, gender, race/ethnicity, education level, and poverty to income ratio. Model 3: Fully adjusted model was adjusted by age, gender, race/ethnicity, education level, poverty income ratio, ALT, AST, SCr, total cholesterol, triglycerides, LDL-C, HDL-C, serum uric acid, albumin, glycohemoglobin, eGFR, BMI, SBP, DBP, waist circumference, physical activity (MET-based rank), current cigarette use, now taking prescribed medicine for HBP, now taking prescribed medicine for high cholesterol level, had at least 12 alcohol drinks/1 year, hypertension history, NAFLD, diabetes history, coronary heart disease

**Table 3:** Multivariate weighted regression model analysis reveals the associations between TFAs (categorical variables) and ACR

Exposure	Palmitelaidic acid	Vaccenic acid	Elaidic acid	Linolelaidic acid	Sum TFAs
Q1	Reference	Reference	Reference	Reference	Reference
Q2	0.08 (-1.01, 1.17) 0.8870	-0.42 (-1.50, 0.66) 0.4420	0.27 (-0.81, 1.35) 0.6221	0.07 (-1.01, 1.15) 0.8985	0.01 (-1.08, 1.09) 0.9914
Q3	0.12 (-1.03, 1.27) 0.8325	0.18 (-0.94, 1.31) 0.7495	0.71 (-0.44, 1.86) 0.2260	0.68 (-0.46, 1.81) 0.2416	0.09 (-1.06, 1.23) 0.8814
Q4	1.05 (-0.23, 2.33) 0.1085	1.03 (-0.21, 2.27) 0.1032	2.02 (0.76, 3.28) 0.0017	1.26 (-0.05, 2.57) 0.0591	1.46 (0.19, 2.73) 0.0244
P for trend	< 0.001	0.014	< 0.001	< 0.001	< 0.001

Q1–Q4: grouped by quartile according to palmitelaidic acid, elaidic acid, linolelaidic acid and sum TFAs. Model: Fully adjusted model was adjusted by age, gender, race/ethnicity, education level, poverty income ratio, ALT, AST, SCr, total cholesterol, triglycerides, LDL-C, HDL-C, serum uric acid, albumin, glycohemoglobin, eGFR, BMI, SBP, DBP, waist circumference, physical activity (MET-based rank), current cigarette use, had at least 12 alcohol drinks/1 year, now taking prescribed medicine for HBP, now taking prescribed medicine for high cholesterol level, hypertension history, NAFLD, diabetes history, coronary heart disease

In addition, we found age, gender, race, hypertension, AST, SCr, and serum albumin persisted as statistically significant factors in relation to the ACR in the fully adjusted model (Table S2). ACR increased by 2.51 mg/g in female participants (*P* < 0.001) in comparison with male participants. When compared with Mexican American, ACR decreased by 1.46 mg/g in non-Hispanic Black (*P* = 0.033). The ACR decreased by 2.21 mg/g in non-hypertension compared with hypertension (*P* < 0.001). Per unit increase in AST, SCr, and serum albumin, the ACR increased by 0.04 mg/g (P = 0.006), 0.09 mg/g (*P* < 0.001), and 0.39 mg/g (*P* < 0.001), respectively.

### XGBoost algorithm model for machine learning

**Fig. 2:**
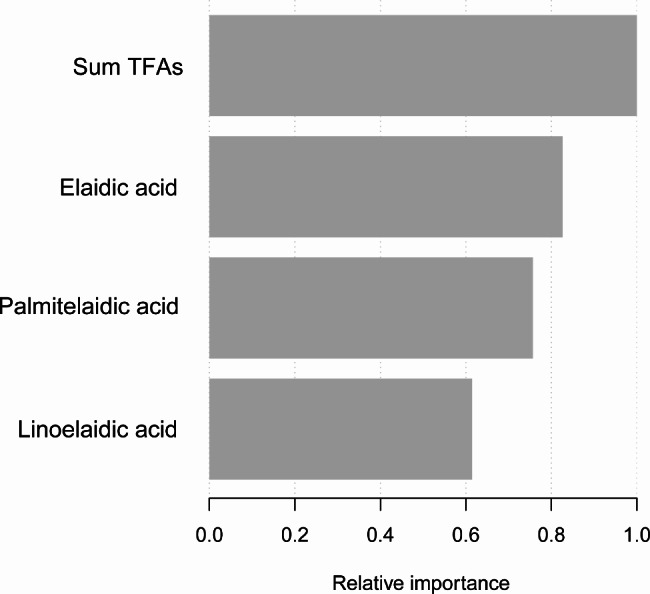
XGBoost model revealed the relative importance of TFAs on the ACR and the corresponding variable importance score. The x-axis showed the importance score, the relative number of a variable used to distribute the data; the y-axis indicated the TFAs.

In order to select the TFAs with the most significant impact on ACR, the XGBoost model was constructed to test the relative importance among circulating TFAs (Fig. 2). Circulating TFAs’ variables included palmitelaidic acid, elaidic acid, linolelaidic acid, and sum TFAs. We discovered that sum TFAs were the most important factor in the ACR, followed by elaidic acid, palmitelaidic acid, and linolelaidic acid. This is consistent with the results of the regression analysis.

### Subgroup analysis between TFAs and the ACR

In Table 4, we conducted an additional analysis of the relationship between TFAs and ACR within a specific subgroup, stratified by factors such as age, BMI, eGFR, hypertension, diabetes, NAFLD, and coronary heart disease. Regarding the subgroup stratified by hypertension and diabetes, a statistically significant positive association was observed with palmitelaidic acid, elaidic acid, linolelaidic acid, and sum TFAs group with hypertension and no diabetes (both *P* < 0.05). Moreover, The ACR of participants with hypertension increased by 1.62 mg/g for each additional unit of log_2_-palmitelaidic acid, increased by 1.71 mg/g for each additional unit of log_2_-elaidic acid, increased 2.00 mg/g for each additional unit of log_2_-linolelaidic acid and increased by 1.79 mg/g for each additional unit of log_2_-sum TFAs (all *P* < 0.05). Additionally, when stratified analysis was performed with hypertension, the interaction test showed that the positive correlation between TFAs and ACR was significantly dependent on hypertension (all *P* for interaction < 0.05).

**Table 4:** Subgroup analysis of TFAs on ACR in the prespecified and exploratory subgroup

	Palmitelaidic acid	Elaidic acid	Linolelaidic acid	Sum TFAs
Stratified by Age				
< 60	0.30 (-0.38, 0.97) 0.390	0.38 (-0.12, 0.88) 0.136	0.44 (-0.24, 1.11) 0.204	0.20 (-0.36, 0.76) 0.486
≥ 60	0.96 (-0.54, 2.45) 0.211	1.39 (0.23, 2.56) 0.019	1.64 (0.04, 3.24) 0.044	1.44 (0.14, 2.74) 0.031
P for interaction	0.186	0.035	0.123	0.023
Stratified by BMI				
< 25	0.32 (-0.75, 1.39) 0.555	0.66 (-0.16, 1.49) 0.116	0.77 (-0.43, 1.98) 0.210	0.69 (-0.21, 1.60) 0.135
25–30	0.43 (-0.68, 1.55) 0.449	0.53 (-0.31, 1.37) 0.214	0.72 (-0.38, 1.81) 0.199	0.29 (-0.65, 1.23) 0.543
> 30	0.74 (-0.56, 2.04) 0.267	0.65 (-0.34, 1.64) 0.196	0.78 (-0.52, 2.09) 0.238	0.60 (-0.50, 1.71) 0.285
P for interaction	0.382	0.478	0.488	0.473
Stratified by eGFR				
< 60	1.69 (-4.81, 8.19) 0.612	0.83 (-4.56, 6.23) 0.763	2.00 (0.40, 3.60) 0.015	1.79 (0.47, 3.11) 0.008
≥ 60	0.07 (-0.58, 0.73) 0.826	0.09 (-0.41, 0.60) 0.720	0.12 (-0.56, 0.80) 0.736	-0.08 (-0.65, 0.48) 0.767
P for interaction	0.143	0.069	0.042	0.033
Hypertension				
Yes	1.62 (0.06, 3.18) 0.042	1.71 (0.53, 2.90) 0.005	2.00 (0.40, 3.60) 0.015	1.79 (0.47, 3.11) 0.008
No	-0.09 (-0.74, 0.57) 0.797	0.09 (-0.41, 0.60) 0.720	0.12 (-0.56, 0.80) 0.736	-0.08 (-0.65, 0.48) 0.767
P for interaction	0.007	0.005	0.013	0.003
Diabetes				
Yes	-3.15 (-6.60, 0.30) 0.075	-0.83 (-3.39, 1.73) 0.526	-0.97 (-4.54, 2.60) 0.595	-1.64 (-4.45, 1.18) 0.255
No	0.80 (0.13, 1.46) 0.019	0.80 (0.30, 1.30) 0.002	0.78 (0.10, 1.46) 0.025	0.78 (0.22, 1.34) 0.007
Border line	5.95 (-2.17, 14.07) 0.165	0.37 (-5.74, 6.49) 0.905	4.67 (-3.60, 12.95) 0.280	1.48 (-5.30, 8.27) 0.673
P for interaction	< 0.001	0.004	0.003	< 0.001
NAFLD				
Yes	0.39 (-1.66, 2.44) 0.709	0.93 (-0.62, 2.47) 0.241	0.99 (-1.22, 3.19) 0.380	0.82 (-0.95, 2.59) 0.362
No	0.69 (-0.02, 1.39) 0.057	0.71 (0.17, 1.24) 0.009	0.82 (0.11, 1.54) 0.024	0.65 (0.06, 1.24) 0.032
P for interaction	0.596	0.853	0.844	0.832
Coronary heart disease				
Yes	-1.96 (-7.76, 3.84) 0.510	2.09 (-2.58, 6.76) 0.383	4.88 (-1.61, 11.38) 0.144	1.42 (-3.86, 6.70) 0.599
No	0.76 (0.09, 1.43) 0.025	0.74 (0.23, 1.24) 0.004	0.77 (0.09, 1.45) 0.026	0.69 (0.13, 1.25) 0.016
P for interaction	0.065	0.486	0.832	0.283

Note1: Model 3: Fully adjusted model was adjusted by age, gender, race/ethnicity, education level, poverty income ratio, ALT, AST, SCr, total cholesterol, triglycerides, LDL-C, HDL-C, serum uric acid, albumin, glycohemoglobin, eGFR, BMI, SBP, DBP, waist circumference, physical activity (MET-based rank), current cigarette use, now taking prescribed medicine for HBP, now taking prescribed medicine for high cholesterol level, had at least 12 alcohol drinks/1 year, hypertension history, NAFLD, diabetes history, coronary heart disease. Note2: In each case, the model was not adjusted for the stratification variable itself. Bold values mean statistically significant

### Non-linearity and threshold effects analysis between TFAs and ACR

**Fig. 3:**
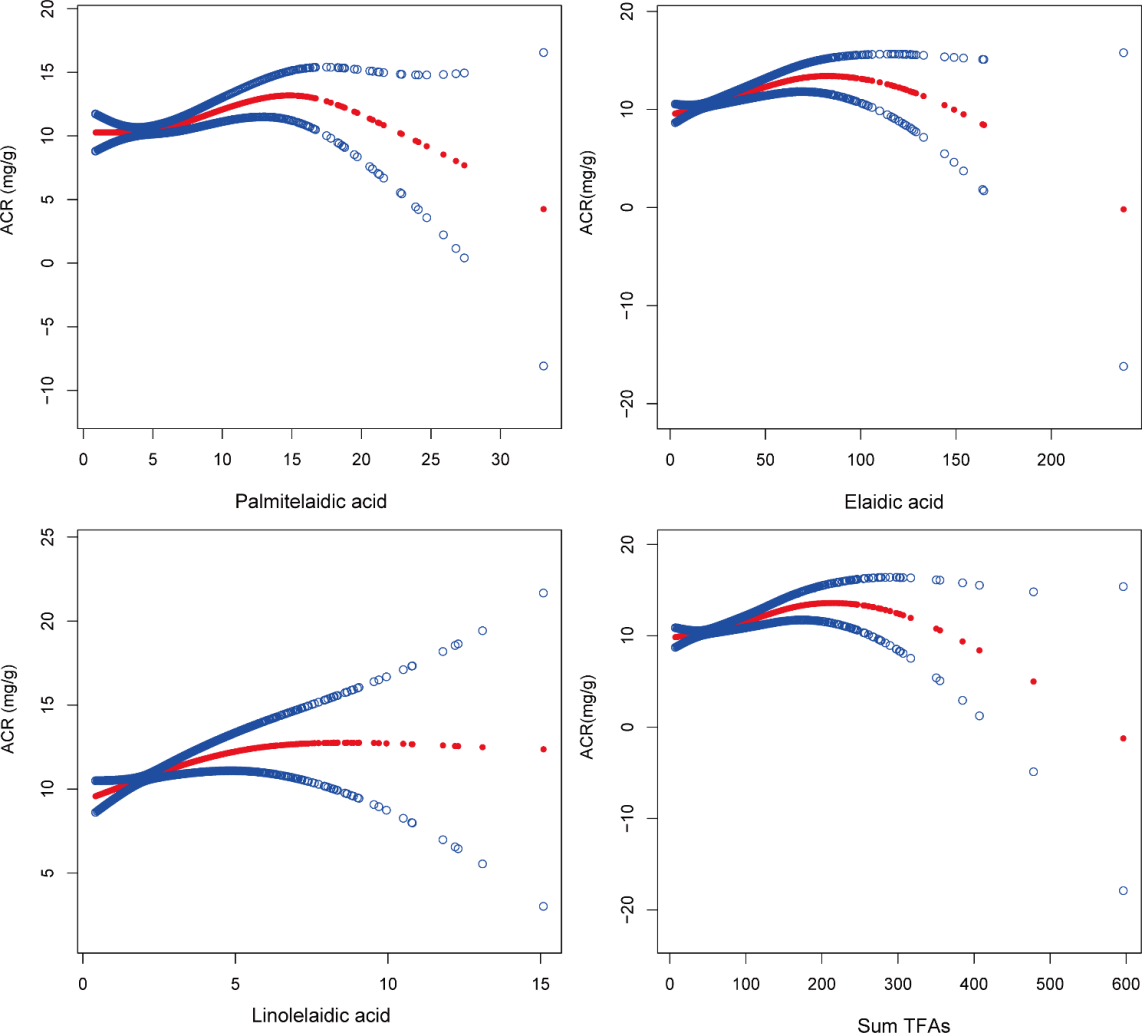
Smooth curve fitting for TFAs and ACR on the fully adjusted model. Non-linear relationship between TFAs and ACR was detected by the generalized additive model. The solid red line represented the smooth fitting curve between variables, and the blue band represented the 95% CI of the fitting

For constructing the non-linear association between the TFAs and ACR, the GAM sensitivity analysis was applied to construct a smooth curve fit after full adjustment since the generalized linear model is incapable of addressing nonlinearity (Fig. 3). Sensitivity analysis of the GAM model supported this positive correlation between TFAs and the ACR, consistent with the results of the multivariable linear regression model. Moreover, the threshold effect is analyzed. The threshold effect value between elaidic acid and ACR was 59.1 µmol/L in selective participants. After full adjustment, the effect value β and 95%CI on the left side of the break point (K) were 0.06 (0.03, 0.09), respectively (*P* = 0.0003). Moreover, there is significant difference in the effect values on the left and right sides of the K, and the logarithmic likelihood ratio test *P* is 0.020 (Table 5).

**Table 5:** Threshold effect analysis of TFAs on ACR using a two-piecewise linear regression model

Exposure:	Palmitelaidic acid	Vaccenic acid	Elaidic acid	Linolelaidic acid	Sum TFAs
two-piecewise linear model					
Break point (K)	11.2	10.6	59.1	4.79	137.86
β1(95% CI), <K P-value	0.26 (0.07, 0.46) 0.0067	-0.43 (-0.91, 0.05) 0.0804	0.06 (0.03, 0.09) 0.0003	0.62 (0.16, 1.08) 0.0089	0.02 (0.01, 0.04) 0.0027
β2(95% CI), >K P-value	-0.15 (-0.55, 0.25) 0.4659	0.03 (0.00, 0.05) 0.0194	-0.03 (-0.08, 0.03) 0.3859	-0.29 (-1.19, 0.61) 0.5335	-0.00 (-0.03, 0.02) 0.7112
β2-β1	-0.41 (-0.89, 0.06) 0.0881	0.45 (-0.03, 0.94) 0.0650	-0.09 (-0.16, -0.01) 0.0208	-0.90 (-1.96, 0.16) 0.0946	-0.03 (-0.06, 0.00) 0.0859
logarithmic likelihood ratio test P-value	0.086	0.064	0.020	0.093	0.084

Fully adjusted model was adjusted by age, gender, race/ethnicity, education level, poverty income ratio, ALT, AST, SCr, total cholesterol, triglycerides, LDL-C, HDL-C, serum uric acid, albumin, glycohemoglobin, eGFR, BMI, SBP, DBP, waist circumference, physical activity (MET-based rank), current cigarette use, now taking prescribed medicine for HBP, now taking prescribed medicine for high cholesterol level had at least 12 alcohol drinks/1 year, smoked at least 100 cigarettes in life, hypertension history, NAFLD, diabetes history, coronary heart disease

### Sensitivity analysis

First, the distribution of characteristics from NHANES (2009–2010, 1999–2000) stratified by ACR stages for selective participants is shown in Table S3. Palmitelaidic acid, vaccenic acid, elaidic acid, linolelaidic acid, and sum TFAs showed distribution differences with statistical significance (all *P* < 0.05). Second, the relationship between TFAs (divided into four quartile ratios) and ACR was analyzed separately for the two NHANES survey cycles 2009–2010 and 1999–2000 (Table S4 and S5). Compared with the lowest corresponding quartile, the ACR increased in all the highest quartiles in the 1999–2000 survey cycle (all *P* for trend < 0.05). In the 2009–2010 survey cycle, the ACR increased in the highest elaidic acid, linolelaidic acid and sum TFAs quartile when compared with the lowest corresponding quartile. Additionally, differences with no statistical significance were observed in the palmitelaidic acid and vaccenic acid quartile. Third, after eliminating the potential effect of extreme values, the smooth curve fitting still supports a positive association between palmitelaidic acid, elaidic acid, linolelaidic acid, sum TFAs and ACR (Figure S1).

## Discussion

As far as we know, our large-scale cross-sectional study is the initial attempt to investigate the latent association between circulating TFA isoforms and the ACR in the United States. With 3785 NHANES participants enrolled, we observed a positive correlation between elaidic acid, linolelaidic acid, sum TFAs, and the ACR in fully adjusted model. More importantly, these associations were more pronounced in the hypertensive/no diabetes population. The XGBoost model for machine learning was built to determine the relative contribution of circulating TFAs to the ACR. It was found that the sum of TFAs was the most critical variable. Furthermore, to assess the non-linearity of the relationship, smooth curve fitting and a threshold effect analysis were constructed. Finally, to verify the robustness of the results, sensitivity analysis was performed.

Since the 1990s, evidence of TFAs being harmful to physical health has been accumulating [36, 37]. In 1999, a rule was proposed by the FDA on TFA labeling, which required that the amount of TFAs per serving should be displayed on packaged foods. After the rule was presented, the intake of TFAs fell from 4.6 g to 1.0 g/person/day from 2003 to 2012 [38]. Industrially-produced trans fats are effectively banned in the U.S., but they remain a major problem in less developed countries. Therefore, our study has important clinical implications for clinical diet modification.

A few studies have determined the effect of TFA intake on renal function; for example, Abbate [24] demonstrated that the ACR was significantly correlated with TFA intake in patients with NAFLD and metabolic syndrome, and Lin [39] demonstrated a direct association between TFA intake and decreased eGFR in models adjusted for age and energy. All these results are in agreement with our results to some extent. However, in these studies, estimates of TFA intake may be limited by recall bias of the food frequency questionnaire, particularly the inability to identify the effects of specific TFA isoforms. Only the sum of all TFA intake was used in the analyses. Besides, studies were restricted to specific populations. Therefore, our study was the first to demonstrate that the different circulating TFA isoforms were positively and independently correlated with urinary albumin excretion in a fully adjusted model.

In our study, most of the participants had ACR levels within the normal range (< 10 mg/g), and almost all the participants had an ACR level of less than 30 mg/g. Previous research evidence suggested that albuminuria, even if the ACR level was less than 30 mg/g, was linked to cardiovascular risk factors and metabolic syndrome in the general population [40]. There is also evidence that ACR levels, which were less than 30 mg/g, had a linear relationship with all-cause mortality [2]. In particular, the ACR has been used to demonstrate the association of even low levels of albuminuria (sub microalbuminuria) with renal events [41]. Another study concluded that any degree of albuminuria (such as microalbuminuria) is a risk factor for cardiovascular events, and in particular, an increase of 3.52 mg/g in the ACR was linked to a 5.9% rise in the risk of CV [42]. Therefore, although the ACR levels of our participants were less than 30 mg/g and the effect size was small, the results still indicate that high levels of circulating TFAs may increase the risk of cardiovascular disease and renal events, so regular screening of urinary albumin and early intervention are warranted. Moreover, in our study, extremely high levels of palmitelaidic acid, elaidic acid, and sum TFAs appear to be negatively associated with the ACR. One reason for this result may be due to inadequate clinical samples. In order to ensure the consistency of the findings, we conducted a sensitivity analysis by excluding the extreme TFA values. Despite this adjustment, the smooth curve fitting continued to demonstrate favorable correlations between palmitelaidic acid, elaidic acid, linolelaidic acid, sum TFAs and the ACR. Therefore, further investigation is required through extensive retrospective and prospective studies involving larger sample sizes to elucidate the associations between different TFA isoforms and the ACR.

Due to the design of the cross-sectional study, we were unable to determine causality. There are several possible causal relationships: (1) High circulating TFAs could cause a high ACR. (2) High ACR could cause high TFA levels. (3) Other factors could cause both high ACR and high TFAs levels. (4) The relationship could be coincidental. It is commonly believed that elevated levels of circulating TFAs may lead to an increased ACR. One possible mechanism by which TFAs increase urinary albumin excretion is through the activation of inflammation. Growing evidence suggests that the consumption of TFAs is associated with dyslipidemia and the activation of systemic inflammatory responses, such as elevated CRP, interleukin-6, and tumor necrosis factor [43]. During a randomized controlled trial involving healthy men, it was observed that consuming a diet containing 8% of daily energy from industrial TFAs resulted in a 3.4-fold rise in plasma concentrations of CRP after 5 weeks of intake, compared to a control diet that did not contain any TFAs [44]. Coincidentally, previous studies have reported a positive correlation between the systemic immune-inflammation index (SII) and urinary albumin excretion in United States adults [29]. Furthermore, a cross-sectional study including 4,926 participants in Wisconsin discovered that inflammatory markers (such as IL-6 levels, TNF-alpha R2, high-sensitivity CRP, and WBC counts) were associated with prevalent CKD [45]. In addition, based on several animal studies, inflammation has been suggested to be associated with decreased renal function and the development of CKD. Tomosugi’s study showed that the pretreatment of rats with TNF and IL-1 increased the severity of glomerular injury in nephritis [46]. Another animal study showed that TNF-α could result in renal dysfunction as well as glomerulosclerosis through enhanced glomerular oxidative stress in obese mice [47]. Therefore, we hypothesized that TFAs cause CKD through inflammation or oxidative stress, as manifested by increased urinary albumin excretion. However, the exact mechanism is still unclear, and more in-depth mechanistic studies are needed.

Furthermore, in a subgroup analysis, we observed significant relationships between the different circulating TFAs and urinary albumin excretion in hypertensive/no diabetic participants. Previous studies have demonstrated that TFA intake contributes to the development of hypertension [48]. Evidence suggests that hypertension, a risk factor for CKD, is linked to an increase in albuminuria [49]. In addition, the associations between the different TFAs and the ACR in our study were also significant in nondiabetic participants. In a prior study, it was found that among populations with limited risk factors, like individuals without diabetes, there was a documented connection between low-grade albuminuria and the risk of all-cause mortality [50]. The results of those studies were all consistent with our conclusion. Collectively, these findings suggest that it is necessary to carefully monitor for increased urinary albumin excretion in individuals with high levels of circulating TFAs, particularly those with hypertension and those without diabetes.

Furthermore, there is heterogeneity in the relationship between circulating TFA isoforms and prognosis [51]. Several studies have shown that elaidic acid may be the major adverse component of TFA intake and that reducing exposure to these isoforms would reduce the deleterious effects of TFA consumption [15, 52]. Analysis of processed food composition based on the GC method showed that elaidic acid was the highest TFA in bakery products, semisolid fats, instant products, and fried potato products [10]. Furthermore, the correlation between elaidic acid and the ACR was statistically significant in all models examined in our study, which aligns with the results from a previous study [43], indicating a positive correlation between total mortality and elaidic acid rather than vaccenic acid.

## Strengths and limitations

This study has several strengths. First, because humans do not synthesize TFAs, circulating TFA subtypes circumvent the recall bias of the food frequency questionnaire. It can reveal dietary intake over the past 6–12 weeks stably. Second, in most previous studies, the relationship between the sum of all TFAs and the ACR was determined, without distinguishing between individual trans isoforms. Third, our results became more dependable following the adjustment for variables that could influence the outcomes. Fourth, subgroup analyses showed that the association between the different TFAs and the ACR were stronger and more significant in those with hypertension and those without diabetes. Therefore, attention should be given to dietary intake and screening of urinary albumin in individuals with high levels of circulating TFAs, particularly those with hypertension and those without diabetes. Nevertheless, the limitations of the study still remain. First, due to the design of the cross-sectional study, the causality relationships could not be determined. Second, the study population was limited to Americans; therefore, generalization is geographically limited. Third, only four plasma isoforms of TFA were identified, while the prognostic significance of the other isoforms remains unclear. Fourth, the sample size was insufficient, and more extensive prospective studies are still needed.

## Conclusion

Our study demonstrated that circulating TFAs are positively and independently correlated with urinary albumin excretion, especially among participants with hypertension or without diabetes. Although most of our participants of ACR levels are less than 30 mg/g, high levels of circulating TFAs may still increase the risk of cardiovascular disease and renal events. Therefore, these findings suggest that it is necessary to carefully monitor for increased urinary albumin excretion in individuals with high levels of circulating TFAs, particularly those with hypertension and those without diabetes.

### Electronic supplementary material

Below is the link to the electronic supplementary material.

Supplementary Material 1

Supplementary Material 2

Supplementary Material 3

Supplementary Material 4

Supplementary Material 5

Supplementary Material 6

## Data Availability

The website below, https://www.cdc.gov/nchs/nhanes/, contains all the available data.

## Abbreviations

TFAs: Trans-fatty acids
ACR: Albumin-creatinine ratio
NHANES: National Health and Nutrition Examination Survey
AST: Aspartate transaminase
ALT: Alanine transaminase
SCr: Serum creatinine
HDL-C: High-density lipoprotein
LDL-C: Low-density lipoprotein
BMI: Body mass index
eGFR: Estimated glomerular filtration rate
DBP: Diastolic blood pressure
SBP: Systolic blood pressure
NAFLD: Non-alcoholic fatty liver disease
