# Supplementary figures and images for: Positive associations between different circulating trans fatty acids (TFAs) and urinary albumin excretion among adults in the U.S.: a population-based study
Source: Lipids Health Dis. 2023 Sep 14;22:152. doi: 10.1186/s12944-023-01917-w (PMC10500873; doi:10.1186/s12944-023-01917-w)

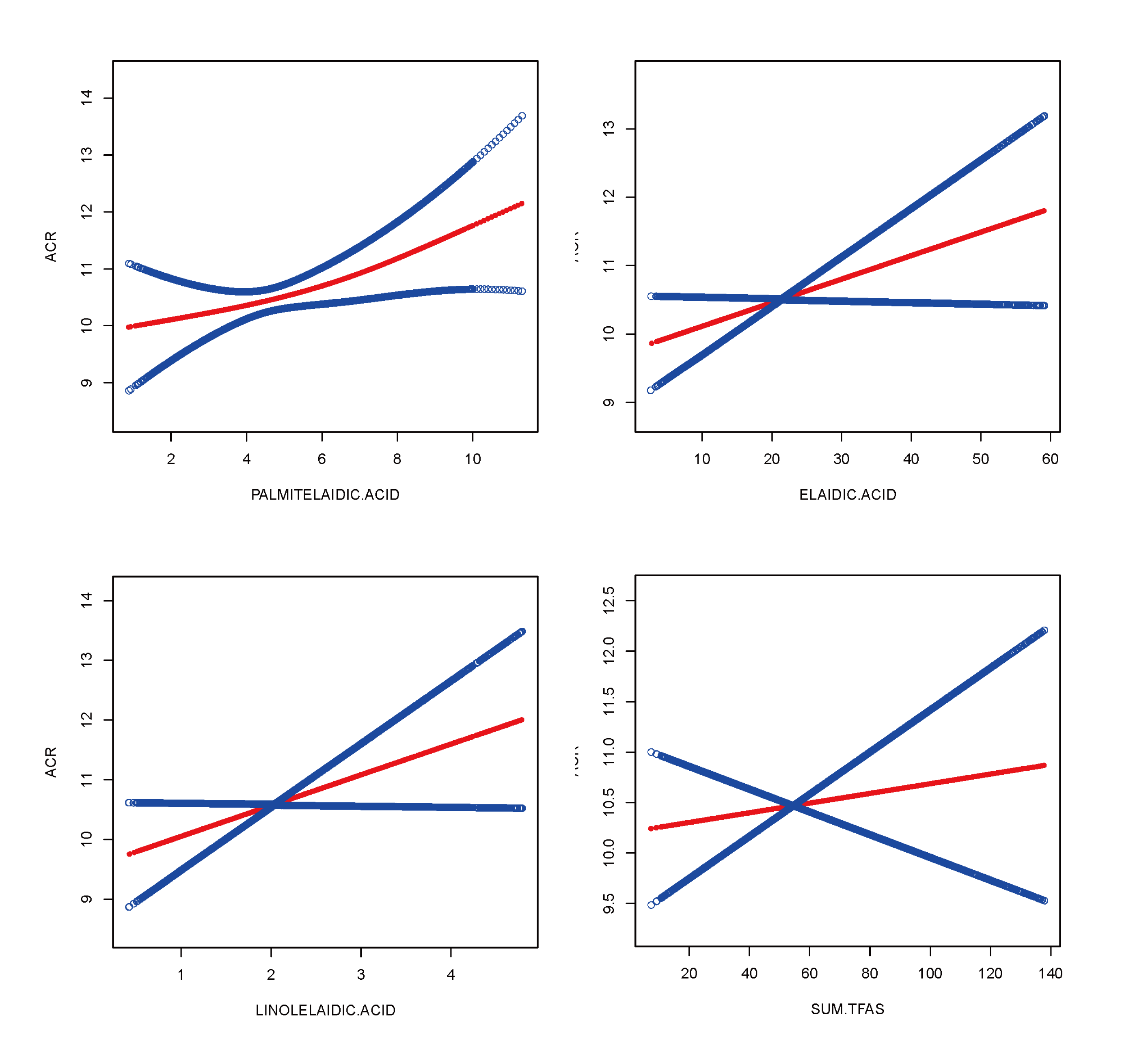

Supplement: Supplementary file 1 — Supplementary Material 1 [file 12944_2023_1917_MOESM1_ESM.png]
