# Supplementary material for: Positive associations between different circulating trans fatty acids (TFAs) and urinary albumin excretion among adults in the U.S.: a population-based study
Source: Lipids Health Dis. 2023 Sep 14;22:152. doi: 10.1186/s12944-023-01917-w (PMC10500873; doi:10.1186/s12944-023-01917-w)
Supplement: Supplementary file 2 — Supplementary Material 2 [file 12944_2023_1917_MOESM2_ESM.docx]

| Exposure | model 1 | model 2 | model 3 |
| --- | --- | --- | --- |
| Palmitelaidic acid | -2.64 (-4.07, -1.20)  0.0003 | 0.37 (-0.82, 1.55) 0.5453 | -2.39 (-3.35, -1.44)  <0.0001 |
| vaccenic acid | -0.59 (-1.80, 0.61)  0.3336 | 0.77 (-0.21, 1.74) 0.1240 | -2.32 (-3.08, -1.57)  <0.0001 |
| Elaidic acid | -0.26 (-1.37, 0.84)  0.6393 | 1.02 (0.11, 1.93) 0.0279 | -2.29 (-3.01, -1.57)  <0.0001 |
| Linolelaidic acid | -1.63 (-3.05, -0.21)  0.0242 | 0.41 (-0.75, 1.57) 0.4888 | -3.74 (-4.71, -2.77)  <0.0001 |
| Sum TFAs | -0.66 (-1.89, 0.57)  0.2951 | 0.91 (-0.09, 1.92) 0.0754 | -2.62 (-3.42, -1.81)  <0.0001 |

**Table S1: Multivariate weighted linear model analysis reveals the association between the log_2_-transformed TFAs and eGFR.**

Model 1: Non-adjusted model adjusts for none. Model 2: Minimally adjusted model adjusts for age, gender, race/ethnicity, education level, and poverty to income ratio. Model 3: Fully adjusted model was adjusted by age, gender, race/ethnicity, education level, poverty income ratio, ALT, AST, SCr, total cholesterol, triglycerides, LDL-C, HDL-C, serum uric acid, albumin, glycohemoglobin, BMI, SBP, DBP, waist circumference, physical activity (MET-based rank), current cigarette use, now taking prescribed medicine for HBP, now taking prescribed medicine for high cholesterol level, had at least 12 alcohol drinks/1 year, hypertension history, NAFLD, diabetes history, coronary heart disease.
