# Supplementary material for: Positive associations between different circulating trans fatty acids (TFAs) and urinary albumin excretion among adults in the U.S.: a population-based study
Source: Lipids Health Dis. 2023 Sep 14;22:152. doi: 10.1186/s12944-023-01917-w (PMC10500873; doi:10.1186/s12944-023-01917-w)
Supplement: Supplementary file 3 — Supplementary Material 3 [file 12944_2023_1917_MOESM3_ESM.docx]

**Table S2 Multivariate linear regression models of ACR.**

| variables | β, (95%CI) | P-value |
| --- | --- | --- |
| Age (years) | 0.15 (0.12, 0.18) | <0.001 |
| Female (versus male) | 2.51 (1.43, 3.58) | <0.001 |
| Race/Ethnicity (versus Mexican American) |  |  |
| Other Hispanic | -1.40 (-2.91, 0.11) | 0.070 |
| Non-Hispanic White | -0.27 (-1.39, 0.85) | 0.639 |
| Non-Hispanic Black | -1.46 (-2.81, -0.12) | 0.033 |
| Other race/ethnicity | 1.19 (-0.90, 3.27) | 0.264 |
| Education level (versus less than high school ) |  |  |
| High school or GED | -0.19 (-1.30, 0.91) | 0.735 |
| Above high school | -0.28 (-1.30, 0.74) | 0.593 |
| Physical activity (versus no physical activity) |  |  |
| Low-intensity | 0.07 (-0.83, 0.97) | 0.876 |
| High intensity | -0.17 (-1.21, 0.86) | 0.743 |
| Had at least 12 alcohol drinks/1 year? (no versus yes) | 0.44 (-0.48, 1.36) | 0.345 |
| Hypertension history (no versus yes) | -2.21 (-3.14, -1.28) | <0.001 |
| NAFLD (no versus yes) | 0.71 (-0.53, 1.95) | 0.262 |
| ALT (IU/L) | -0.02 (-0.04, 0.01) | 0.232 |
| AST (IU/L) | 0.04 (0.01, 0.07) | 0.006 |
| SCr (μmol/L) | 0.09 (0.06, 0.12) | <0.001 |
| Total Cholesterol (mmol/L) | -0.46 (-1.96, 1.05) | 0.553 |
| Triglyceride (mmol/L) | 0.47 (-0.16, 1.09) | 0.145 |
| LDL-C (mmol/L) | -0.10 (-1.68, 1.47) | 0.896 |
| HDL-C (mmol/L) | 0.94 (-0.88, 2.75) | 0.311 |
| Serum albumin (g/L) | 0.39 (0.25, 0.53) | <0.001 |
| BMI, mean ± SD (Kg/m^2^) | -0.08 (-0.22, 0.06) | 0.250 |

The unit for continuous variables and the reference group for categorical variables are provided next to the variables. The β of ACR was each unit increase in continuous variables and compared with the reference group for categorical variables.
