# Supplementary material for: Positive associations between different circulating trans fatty acids (TFAs) and urinary albumin excretion among adults in the U.S.: a population-based study
Source: Lipids Health Dis. 2023 Sep 14;22:152. doi: 10.1186/s12944-023-01917-w (PMC10500873; doi:10.1186/s12944-023-01917-w)
Supplement: Supplementary file 4 — Supplementary Material 4 [file 12944_2023_1917_MOESM4_ESM.docx]

|  | ACR<10mg/g | 10≤ACR<30mg/g | ACR≥30mg/g | P-value |
| --- | --- | --- | --- | --- |
| N | 2677 | 835 | 273 |  |
| Palmitelaidic acid | 5.31 ± 2.92 | 5.70 ± 3.44 | 6.09 ± 3.43 | <0.001 |
| vaccenic acid | 28.53 ± 19.95 | 30.28 ± 23.13 | 32.90 ± 23.97 | 0.011 |
| Elaidic acid | 23.38 ± 17.70 | 26.34 ± 20.64 | 28.56 ± 20.73 | <0.001 |
| Linolelaidic acid | 2.18 ± 1.28 | 2.40 ± 1.51 | 2.47 ± 1.40 | <0.001 |
| Sum TFAs | 59.40 ± 40.50 | 64.72 ± 47.38 | 70.01 ± 48.13 | <0.001 |

**Table S3: Difference in TFAs across stages of ACR in NHANES 2009-2010 and 1999-2000**

Abbreviations: TFAs, trans fatty acids; ACR: urine albumin-to-creatinine ratio; Data are presented as mean ± standard deviation; *P* obtained by an ANOVA test for continuous variables.
