# Supplementary material for: Positive associations between different circulating trans fatty acids (TFAs) and urinary albumin excretion among adults in the U.S.: a population-based study
Source: Lipids Health Dis. 2023 Sep 14;22:152. doi: 10.1186/s12944-023-01917-w (PMC10500873; doi:10.1186/s12944-023-01917-w)
Supplement: Supplementary file 5 — Supplementary Material 5 [file 12944_2023_1917_MOESM5_ESM.docx]

**Table S4: Multivariate weighted regression model analysis reveals the associations between TFAs (categorical variables) and ACR in NHANES 2009-2010**

| Exposure | Palmitelaidic acid | Vaccenic acid | Elaidic acid | Linolelaidic acid | Sum TFAs |
| --- | --- | --- | --- | --- | --- |
| Q1 | Reference | Reference | Reference | Reference | Reference |
| Q2 | 0.67 (-0.66, 2.00) 0.3223 | 0.84 (-0.48, 2.16) 0.2107 | 0.46 (-0.87, 1.79) 0.4960 | 0.64 (-0.68, 1.96) 0.3426 | 0.27 (-1.06, 1.59) 0.6943 |
| Q3 | 0.35 (-1.03, 1.73) 0.6193 | -0.09 (-1.44, 1.26) 0.8967 | 0.96 (-0.42, 2.35) 0.1730 | 0.07 (-1.32, 1.47) 0.9174 | 1.04 (-0.33, 2.42) 0.1361 |
| Q4 | 1.67 (0.12, 3.22) 0.0346 | 1.43 (-0.05, 2.90) 0.0576 | 2.78 (1.20, 4.36) 0.0006 | 2.09 (0.51, 3.67) 0.0096 | 1.62 (0.07, 3.16) 0.0400 |
| P for trend | 0.072 | 0.183 | <0.001 | 0.036 | 0.024 |

Q1–Q4: grouped by quartile according to palmitelaidic acid, elaidic acid, linolelaidic acid and sum TFAs. Model: Fully adjusted model was adjusted by age, gender, race/ethnicity, education level, poverty income ratio, ALT, AST, SCr, total cholesterol, triglycerides, LDL-C, HDL-C, serum uric acid, albumin, glycohemoglobin, eGFR, BMI, SBP, DBP, waist circumference, physical activity (MET-based rank), current cigarette use, had at least 12 alcohol drinks/1 year, now taking prescribed medicine for HBP, now taking prescribed medicine for high cholesterol level, hypertension history, NAFLD, diabetes history, coronary heart disease
