# Supplementary material for: Positive associations between different circulating trans fatty acids (TFAs) and urinary albumin excretion among adults in the U.S.: a population-based study
Source: Lipids Health Dis. 2023 Sep 14;22:152. doi: 10.1186/s12944-023-01917-w (PMC10500873; doi:10.1186/s12944-023-01917-w)
Supplement: Supplementary file 6 — Supplementary Material 6 [file 12944_2023_1917_MOESM6_ESM.docx]

**Table S5: Multivariate weighted regression model analysis reveals the associations between TFAs (categorical variables) and ACR in NHANES 1999-2000**

| Exposure | Palmitelaidic acid | Vaccenic acid | Elaidic acid | Linolelaidic acid | Sum TFAs |
| --- | --- | --- | --- | --- | --- |
| Q1 | Reference | Reference | Reference | Reference | Reference |
| Q2 | 0.16 (-1.66, 1.99) 0.8613 | 0.06 (-1.73, 1.85) 0.9510 | -0.51 (-2.31, 1.30) 0.5829 | 0.48 (-1.33, 2.29) 0.6025 | 0.35 (-1.45, 2.15) 0.7049 |
| Q3 | 1.16 (-0.73, 3.05) 0.2302 | 2.13 (0.31, 3.95) 0.0220 | 2.17 (0.31, 4.03) 0.0224 | 1.93 (0.02, 3.84) 0.0482 | 2.13 (0.27, 3.99) 0.0250 |
| Q4 | 3.67 (1.54, 5.79) 0.0007 | 2.72 (0.69, 4.75) 0.0086 | 3.44 (1.33, 5.55) 0.0015 | 3.59 (1.30, 5.88) 0.0021 | 3.03 (0.93, 5.13) 0.0047 |
| P for trend | <0.001 | 0.001 | <0.001 | 0.001 | 0.001 |

Q1–Q4: grouped by quartile according to palmitelaidic acid, elaidic acid, linolelaidic acid and sum TFAs. Model: Fully adjusted model was adjusted by age, gender, race/ethnicity, education level, poverty income ratio, ALT, AST, SCr, total cholesterol, triglycerides, LDL-C, HDL-C, serum uric acid, albumin, glycohemoglobin, eGFR, BMI, SBP, DBP, waist circumference, physical activity (MET-based rank), current cigarette use, had at least 12 alcohol drinks/1 year, now taking prescribed medicine for HBP, now taking prescribed medicine for high cholesterol level, hypertension history, NAFLD, diabetes history, coronary heart disease
